# The Role of Reactive Oxygen Species (ROS) in the Biological Activities of Metallic Nanoparticles

**DOI:** 10.3390/ijms18010120

**Published:** 2017-01-10

**Authors:** Ahmed Abdal Dayem, Mohammed Kawser Hossain, Soo Bin Lee, Kyeongseok Kim, Subbroto Kumar Saha, Gwang-Mo Yang, Hye Yeon Choi, Ssang-Goo Cho

**Affiliations:** Department of Stem Cell & Regenerative Biotechnology, Incurable Disease Animal Model and Stem Cell Institute (IDASI), Konkuk University, Gwangjin-gu, Seoul 05029, Korea; ahmed_morsy86@yahoo.com (A.A.D.); kawsersau07@gmail.com (M.K.H.); soobineey@naver.com (S.B.L.); proproggs@naver.com (K.K.); subbroto@konkuk.ac.kr (S.K.S.); slayersgod@nate.com (G.-M.Y.); hyeon.choi24@gmail.com (H.Y.C.)

**Keywords:** nanoparticles (NPs), reactive oxygen species (ROS), stem cells, toxicity, cellular signaling, regenerative medicine

## Abstract

Nanoparticles (NPs) possess unique physical and chemical properties that make them appropriate for various applications. The structural alteration of metallic NPs leads to different biological functions, specifically resulting in different potentials for the generation of reactive oxygen species (ROS). The amount of ROS produced by metallic NPs correlates with particle size, shape, surface area, and chemistry. ROS possess multiple functions in cellular biology, with ROS generation a key factor in metallic NP-induced toxicity, as well as modulation of cellular signaling involved in cell death, proliferation, and differentiation. In this review, we briefly explained NP classes and their biomedical applications and describe the sources and roles of ROS in NP-related biological functions in vitro and in vivo. Furthermore, we also described the roles of metal NP-induced ROS generation in stem cell biology. Although the roles of ROS in metallic NP-related biological functions requires further investigation, modulation and characterization of metallic NP-induced ROS production are promising in the application of metallic NPs in the areas of regenerative medicine and medical devices.

## 1. Introduction

Reactive oxygen species (ROS) are natural byproducts of cellular oxidative metabolism and play important roles in the modulation of cell survival, cell death, differentiation, cell signaling, and inflammation-related factor production [1,2]. Biologically-significant ROS elements include free radicals, such as singlet oxygen (^1^O_2_), superoxide (O_2_^•–^), hydroxyl (HO^•^), hydroperoxyl (HO_2_^•^), carbonate (CO_3_^•–^), peroxyl (RO_2_^•^), alkoxyl (RO^•^), and carbon dioxide radical (CO_2_^•–^), and nonradicals, such as hydrogen peroxide (H_2_O_2_), hypobromous acid (HOBr), hypochlorous acid (HOCl), ozone (O_3_), organic peroxides (ROOH), peroxynitrite (ONOO^–^), peroxynitrate (O_2_NOO^–^), peroxynitrous acid (ONOOH), peroxomonocarbonate (HOOCO_2_^–^), nitric oxide (NO), and hypochlorite (OCl^–^) [3,4,5].

**Figure 1:**
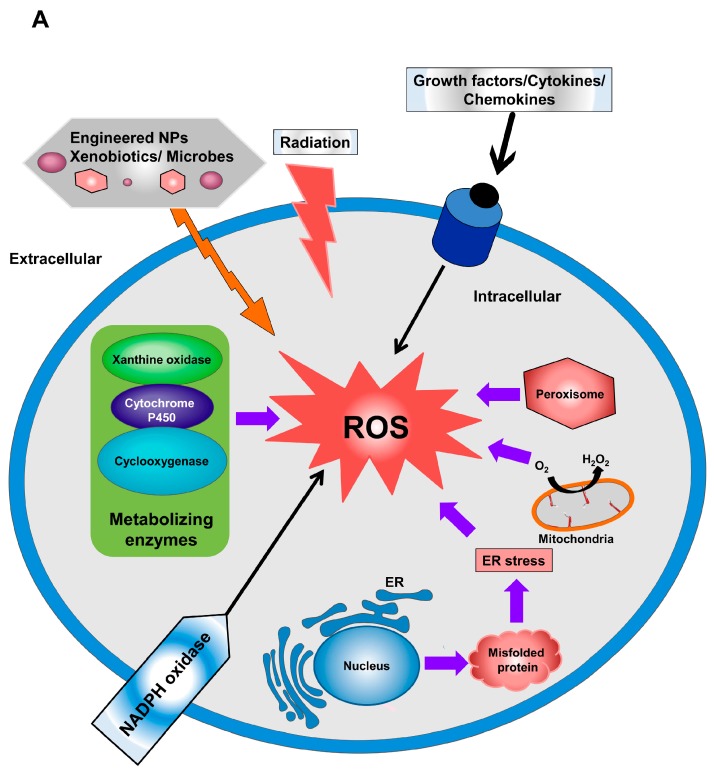

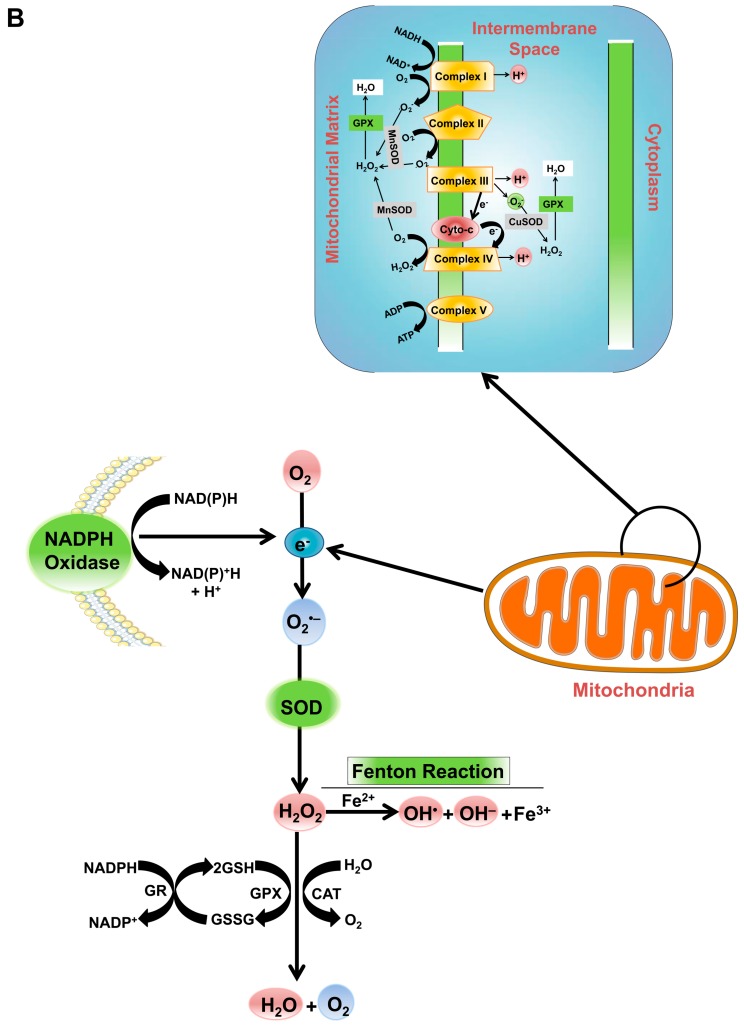
Sources of Reactive oxygen species (ROS) generation. (**A**) Descriptive diagram outlining the extracellular and intracellular sources of ROS generation. The extracellular sources of ROS are represented by environmental pollutants, radiation exposure, microbial infection, and exposure to engineered Nanoparticles (NPs). Intracellular ROS can be generated from the mitochodria, endoplasmic reticulum (ER) stress, cellular-metabolizing enzymes, and the NOX family; and (**B**) a schematic diagram summarizing the formation of ROS from nicotinamide adenine dinucleotide phosphate (NADPH) oxidase and mitochodria and the mechanisms involved in ROS scavenging of ROS. NOX: NADPH oxidase; SOD: superoxide dismutase; CAT: catalase; GPX: glutathione peroxidase; e^−^: electron; GR: glutathione reductase; Cyto-c: cytochrome c; and GSSG: Glutathione disulfide.

O_2_^•–^ is a free radical with a short biological lifespan attributed to its rapid reduction to H_2_O_2_, which is mediated by superoxide dismutases (SODs) [6]. However, H_2_O_2_ is a non-radical derivative of ROS, with a long biological lifespan and higher stability as compared with free radicals [7]. Mitochondria and nicotinamide adenine dinucleotide phosphate (NADPH) oxidase (NOX) generate superoxide, which can inactivate specific enzymes or initiate lipid peroxidation (Figure 1A) [8]. Incomplete electron reduction of O_2_ leads to superoxide production and ultimately conversion into H_2_O_2_ by SOD, which constitutes a key antioxidant defense present in nearly all cells exposed to oxygen. There are three forms of SOD, including SOD1 (a copper- and zinc-ion-containing SOD primarily located in the cytoplasm), SOD2 (a manganese-ion-containing mitochondrial SOD), and SOD3 (a copper- and zinc-ion-containing extracellular SOD).

Under various physiological states, ROS are produced as intermediates, and their cellular levels are strongly regulated by various detoxifying enzymes, such as SOD, glutathione peroxidase (GPX), and catalase (CAT), or by different antioxidants, including flavonoids, ascorbic acids, vitamin E, and glutathione (GSH) [4]. There are significant correlations between ROS generation and metabolism, as well as with cellular pathophysiology [9,10].

Reduction-oxidation (redox) imbalance represents a defect in the balance between ROS generation and the neutralization of excess ROS by cellular antioxidant factors. Disturbed redox homeostasis leads to harmful effects on cells mediated by interference with cell-signaling mechanisms or resulting in oxidative damage to biomolecules, such as proteins, lipids, and nucleic acids [11]. By contrast, modulation of ROS generation enhances activation of key signaling molecules that regulate cell death, survival, differentiation, and proliferation [12,13,14,15].

Nanotechnology is a branch of science that deals with tiny materials and their surfaces with dimensions <100 nm [16]. This field has rapidly developed in the 21st century, with gradual advances in novel applications. Engineered nanoparticles (NPs) exhibit specific physicochemical characteristics and are manufactured for applications in several biological and commercial functions [17]. The unique biological and chemical, thermal, and electrical characteristics of NPs make them valuable in numerous applications in the areas of commercial industry, agriculture, medicine, cosmetics, clothing, and food [18,19,20]. The absolute diversity of the physicochemical characteristics of NPs also creates research opportunities pertaining to their toxic effects [21].

NPs internalization into the human body can be facilitated via various routes, including inhalation, oral intake, and skin absorption. Following uptake, they are introduced to the biological environment, where they interact with cellular molecules present in body fluids. The protein corona occurs as a result of NP surfaces being coated with cellular molecules [22,23], which enables recognition of NP biological identity [24]. Investigations into the cellular toxicity and phototoxicity of NPs are required for the safe development and use of nanotechnology and commercial NPs, because NP-mediated toxicity can potentially result in inflammation, oxidative stress, genetic damage, inhibition of cell division, and cell death [25,26].

NP-mediated ROS generation initiates a sequence of pathological events, including inflammation, fibrosis, genotoxicity, and carcinogenesis, and is modulated by physicochemical features of NPs, such as size, charge, surface area, and chemical structure [27]. NP-related toxicity can trigger increased expression of pro-inflammatory and fibrotic cytokines and activation of inflammatory cells, such as macrophages and neutrophils, which can influence the enhanced generation of ROS [28,29,30].

The mechanism associated with NP-induced ROS generation varies among different NPs, and the core cellular mechanism related to ROS production remains unexplained. The majority of metal-based NPs may provoke free-radical-facilitated toxicity via Fenton-type reactions [31,32]. Since ROS generation is a key byproduct of NP-induced injury or modulation of cellular function, in this review, we discussed the sources of ROS generation, NPs classes and their biomedical applications, the sources and roles of ROS in NP-related biological functions, and the mechanisms of ROS-mediated biological activities associated with NPs in various cells.

## 2. Sources of Reactive Oxygen Species (ROS) Generation

The main sources of intracellular ROS are mitochondria, the endoplasmic reticulum (ER), peroxisomes, microsomes, and NOX complexes (seven distinct isoforms) in cell membranes (Figure 1A) [33,34]. Specifically, mitochondria represent the main intrinsic source of ROS generation via the mitochondrial electron-transport system (Figure 1B) [35]. Increased accumulation of calcium (Ca^2+^) in the cytoplasm results in activation of the mitochondrial electron-transport chain and ROS generation. During mitochondrial production of adenosine triphosphate (ATP) and water, small concentrations of oxygen are produced, resulting in the early stages of ROS production. The superoxide anion, the first ROS element generated by mitochondria, is produced by complex I (NADH ubiquinone oxidoreductase) and complex III (co-enzyme Q, bc1 complex, and uniquinone/cytochrome c reductase) activity in the mitochondrial matrix and intermembrane space, respectively [36,37]. In the intermembrane space, metals, such as Cu, Mn, and Zn-SOD, catalyze the conversion of superoxide anions into H_2_O_2_ (stable form) [38]. Monoamine oxidase and α-ketoglutarate dehydrogenase are also potential sources of mitochondrial ROS generation [39,40].

NOX represents a non-mitochondrial source of ROS generation and plays pivotal role in superoxide formation via oxygen reduction mediated by the electron donor NADPH. Mammalian NOX is composed of seven isoforms (NOX1-5, Dual oxidase 1 (DUOX1), and DUOX2), the majority of which generate superoxide, whereas NOX4, DUOX1, and DUOX2 generate H_2_O_2_ [41,42].

The ER is a cellular organelle that also plays a key role in ROS production. The ER lumen represents a suitable oxidizing environment (with a high ratio of oxidized-to-reduced forms of GSH) for protein folding and formation of disulfide bonds [43].

Additionally, there are various cellular enzymes, including xanthine oxidoreductase, nitric oxide (NO) synthase, cytochrome P_450_ monoxygenase, lipoxygenase, and cyclooxygenase, implicated in the process of ROS generation. ONOO^–^, which is considered a potent oxidizing and nitrating agent, results from interaction between NO and O_2_^•–^ [44]. 

Extracellular sources of ROS generation include ROS-inducing agents, such as radiation, pollutants, and exposure to nanomaterials (Figure 1A) [45]. Oxidative stress initiates defense strategies associated with macrophages and neutrophils against microbial invasion, cancer, and the exposure to pollutants. Given the role of iron in the Fenton reaction, which is implicated in the formation of hydroxyl radicals, free iron (Fe^2+^) is a critical factor related to toxicity induced by ROS generation [46,47].

## 3. Nanoparticle (NP) Classes and Biomedical Applications

Based on their preparation, NPs can be broadly classified into two main classes: organic NPs, include dendrimers, liposomes, carbon-based nanomaterials, and polymeric micelles, and inorganic NPs, including metal and metal oxides, quantum dots (QDs), and magnetic NPs.

**Figure 2:**
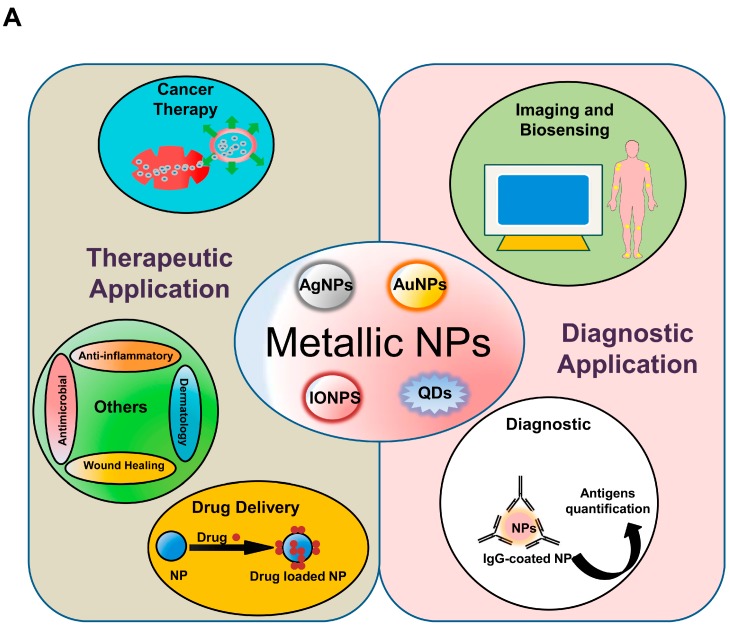

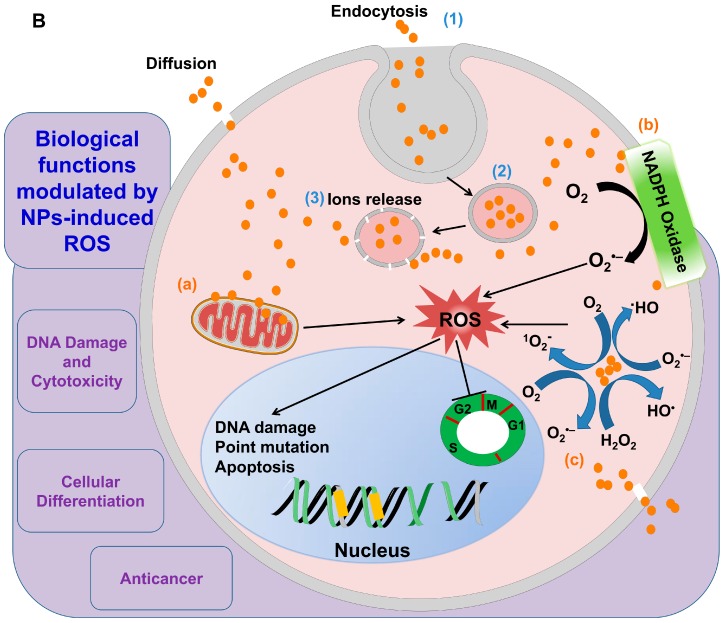
The biomedical applications of metallic NPs and the mechanisms of NP-mediated ROS generation. (**A**) Summary of the nanomaterial applications in the medical field; (**B**) schematic diagram describing the mechanisms implicated in NP-induced ROS production. NPs can be internalized into the cell by (1) endocytosis; (2) formation of the endocytotic vesicles; and (3) release of particle ions from vesicles into the cell. The main factors responsible for ROS generation by NPs include: (**a**) interaction with the mitochodria; (**b**) interaction with NADPH oxidase; and (**c**) factors related to the physicochemical properties (size, shape, photoreactive properties, and surface chemistry). These factors lead to ROS generation and its consequences, including DNA damage, cell cycle arrest, alterations in apoptosis, and damage to the cell membrane.

Metallic NPs can be synthesized and modified with various surface functionalities, which allow them to be conjugated with antibodies, ligands, and drugs, thereby increasing their potential applications in biotechnology, magnetic separation, drug and gene delivery, and imaging. Recently, metallic NPs, such as magnetic NPs (iron-oxide NPs (IONPs)), silver NPs (AgNPs), gold NPs (AuNPs), and QDs, have been consistently used and adapted to enhance their functions as diagnostic and therapeutic agents. These applications are briefly described in subsequent sections (Figure 2A).

### 3.1. Optical Imaging

Fluorescence proteins and luciferase bioluminescence-based techniques commonly used for imaging possess many drawbacks associated with low intensity, low stability, and poor optimization [48]. Fortunately, NP applications offer possibilities to overcome these limitations. In this context, various markers can be detected simultaneously using QDs and based on their high quantum properties [49]. Various reports attributed the broad application of various metallic NPs, such as AgNPs, AuNPs, and aluminum (Al) NPs in the imaging to their plasmonic properties [50,51]. The application of superparamagnetic IONPs (SPIONS) overcomes the low sensitivity of magnetic resonance imaging by boosting the contrast of magnetic imaging [52].

### 3.2. Biosensing

Biosensor is device for detection of biological elements, such as nucleic acids, cells, protein, microorganisms, and enzymes, which ultimately analyze the biological alterations [53]. It mainly composed of biological recognition components (bio-transducer and bio-receptor) [53,54]. Enormous scientific efforts are designated to discover highly sensitive and economical biosensors. NPs can also be applied as sensors for chemicals and biological molecules [55]. For example, the presence of heavy metals, fungal toxins, and microbes in water, as well as the nutritional value of soil and agricultural pests, can be detected and estimated using NPs or nanosensors [56,57]. AuNPs are widely applied in biosensing, which is ascribed to their biocompatibility, unique optical and electric characteristics, and the convenience of their production and surface modifications [58]. AuNPs, in particular, possess surface plasmon resonance properties that allow the electrons to oscillate upon irradiation with single-wavelength light. This oscillation is dependent on the particle size, shape, and the dielectric constant [59]. The alteration on the oscillation and the resulted color led to visual recognition of the changes in the surrounding environment. Accordingly, there are a wide range of nanomaterials as colorimetric biosensors has been emerged for the bio-analysis of DNA or immunity related molecules [60,61,62]. Immunoassays with high sensitivity and specificity were developed using AuNPs for detection of anti-protein A [63], and coating of AuNPs with immunoglobulin G allows for a highly sensitive immunoassay to detect even small amounts of antigens in samples using the hyper-Rayleigh scattering method [64]. However, these two methods are only able to identify proteins at the microgram level, which confines their uses in immunoassays specifically for early cancer diagnosis [65].

### 3.3. Diagnostic Applications

NPs can combine with other specific materials to allow for highly accurate diagnoses at the molecular level [66]. There are various commercial nanomaterials available for medical diagnostic purposes that have been previously reviewed elsewhere [48,67].

The potentials of NPs for use as tags of DNA, proteins, microbes, and other cellular molecules make them promising materials for various diagnostic applications [68,69,70].

### 3.4. Drug Delivery

NPs enable the delivery of various drugs in several biomedical areas. NP-mediated drug delivery is more advantageous than use of conventional methods in terms of their high specificity, low side effects, and cost-effectiveness [71,72]. 

Previous studies reported the ability of NPs to deliver multiple drugs, which is efficient for the treatment of complicated diseases. In this context, loading of DNA oligonucleotides to Au nanorods via thiol conjugation resulted in the efficient release of DNA while maintaining its functionality following release [73]. The release of DNA from the Au nanorods was modulated by ultrafast laser radiation, which selectively melted the nanorods through longitudinal surface-plasmon resonance. The application of AuNPs exhibited highly efficient delivery of the anticancer drug oxaliplatin, which was successfully transported to the nucleus of lung cancer cells without any signs of cytotoxicity [74]. This method obviated the hurdles of dose-associated side effects and previously observed resistance to anticancer drugs.

### 3.5. Other Applications

The unique physicochemical properties of NPs together with their biocompatibility make them ideal for various applications, including in cosmetics, for tissue regeneration, and as antimicrobial, anticancer, anti-inflammatory, and detoxifying agents [75,76]. The potent antimicrobial, anticancer, and wound-healing potential of AgNPs was previously reported [77], with Zn and titanium (Ti) NPs also exhibiting successful applications in dermatology [78,79].

## 4. Mechanisms Associated with NP-Induced ROS Generation

NP-related ROS generation is governed by the following factors: NPs internalization, particle chemistry, and physical properties (size and surface area) [80,81]. The mechanism of NP-induced ROS generation involving NP-related factors implicated in ROS generation and the interaction of NPs with cellular components is described in this section (Figure 2B).

### 4.1. NP-Related Factors Implicated in ROS Generation

The potential of various nanomaterials with different chemical structures to generate ROS associated with their hazardous and toxic effects has been well-characterized in previous studies [45,82]. Compared with microparticles or their bulk of origin, NPs possess unique physicochemical properties (size, surface area, shape, solubility, and aggregation status) that correlate with their potential to generate ROS [83,84,85,86,87,88].

NPs differ significantly from their bulk counterparts in terms of surface area, the latter of which is significantly larger and also contains a higher fraction of atoms [89]. Particle mass, which represents the surface-to-volume ratio, is inversely correlated with particle size [89]. Smaller NPs are associated with larger surface areas and mass; therefore, particle size regulates the number of reactive sites on the NP surface [90,91,92]. Moreover, chemical reactions are significantly accelerated with NPs with larger surface area. The high chemical reactivity of NPs is attributed to the dangling bonds (immobilized free radicals) of the atoms located on the NP surface that promote NP-induced biochemical catalysis [93]. Compared with larger NPs, smaller NPs result in structural modifications and alterations in the electronic properties of the particle surface, ultimately resulting in formation of reactive groups on particle surfaces [94,95]. Previous studies showed that silicon NPs and ZnO NPs with the same size and shape exhibited different degrees of toxicity attributed to differences in their surface properties. For example, ZnO NPs possess higher chemical activity as compared with that shown by silicon oxide (SiO_2_) NPs and, as a consequence, produce higher levels of oxidative stress caused by the production of O_2_^•–^.

Previous reports illustrated the impact of reactive surfaces of NPs in ROS production [45,96]. The oxidants and free radicals located on the particle surface modulate ROS generation. For example, the production of ROS (HO^•^ and O_2_^•–^) from quartz particles is ascribed to the presence of surface-bound radicals, such as SiO^•^ and SiO_2_^•^ [32,81]. Additionally, adsorption of ambient particulate matter, such as ozone and nitrogen oxide, onto the NP surface boosts its potential to generate high levels of oxidative stress [89].

Aqueous suspensions of quartz particles produce H_2_O_2_, HO^•^, and ^1^O_2_ [32,45,96]. Despite surface-dependent properties, chemical compounds and metals present on the NP surface increase the speed of ROS reactions [90]. 

There are various transition metals, including Si, Fe, copper (Cu), chromium (Cr), and vanadium, that are implicated in ROS generation through Haber-Weiss and Fenton reaction mechanisms [81]. In Fenton reactions, a transition metal ion reacts with H_2_O_2_ to yield HO^•^ and an oxidized metal ion. Additionally, the reduction of H_2_O_2_ with ferrous iron (Fe^2+^) also culminates in creation of HO^•^ that is exceedingly sensitive and toxic to biological molecules [34]. 

Metallic NPs, such as Cu and Fe, influence oxidative stress (O_2_^•–^ and HO^•^) via Fenton reactions [97], whereas Haber-Weiss reactions represent the reactions between oxidized metal ions and H_2_O_2_ to produce HO^•^ [34,98]. NPs containing Cr, cobalt (Co), and vanadium (Va) can catalyze both Fenton and Haber-Weiss reactions [97], and Fenton reactions are involved in IONP-induced ROS generation [99].

Quantum confinement effects are also play a critical roles in the unique function of QDs NPs. Nanomaterials exhibiting quantum-confinement effects possess magnetic moments that are absent in original bulks [100]. Quantum confinement also modulates NP affinity to accept or donate electrical charges that, in turn, influence their catalytic behavior [100]. Compared with their original bulk material, atoms located on the NP surface have fewer neighboring atoms that can potentially decrease the binding energy of each atom [100]. As a consequence, NP melting temperature is lower than that of the bulk material according to the Gibbs-Thomson equation [100]. 

Some NPs activated upon exposure to photon energy (ultraviolet/visible irradiation), such as TiO_2_ NPs and QDs, produce electrons [101,102] that possess energy capable of converting O_2_ into ^1^O_2_, which is implicated in cellular damage mediated by interactions with cellular proteins, lipids, and nucleic acids [103].

### 4.2. NP- and Cellular-Component-Induced ROS Generation

In order to invade the cell, NPs need to interact with cell membranes possessing unique properties that modulate the exchange of various ions and molecules from the external environment (Figure 2B). Protein aggregation, lipids, lipoproteins, and nanomaterials can be transported to and from cells via encapsulation within vesicles by endocytosis (transportation into cells) and exocytosis (transportation outside of cells). Endocytosis can be dependent or independent of caveolin or clathrin proteins and plays an important role in cellular internalization of NPs [104,105]. 

Factors involved in the NP-induced intracellular ROS generation include catalysis of free-radical reactions, interaction with mitochondrial components, activation of growth factors, and activation of NOX [106].

Mitochondria represent a key organelle involved in NP-related generation of cellular ROS. The ability of NPs to depolarize the mitochondrial membrane and to interfere with the electron-transport chain through activation of NADPH-related enzymes was previously described [107,108]. The mitochondrial electron-transport chain can be blocked following exposure to NPs, thereby increasing cellular levels of O_2_^•–^ via electron transfer from respiratory carriers to O_2_ [106]. AgNP-exposed human glioblastoma and human fibroblast cells showed increased accumulation of AgNPs in mitochondria that led to disruption of the mitochondrial electron-transfer chain and, consequently, high levels of ROS-mediated cytotoxicity [109]. The interaction of Ag ions with NADH dehydrogenase, which blocks electron transfer to O_2_ and generation of high levels of ROS, was shown in *Escherichia coli* [110]. Additionally, NP exposure leads to activation of immune cells in an ROS-dependent mechanism, which is mediated by NOX activation [80].

NP-induced production of free radicals leads to reduction of GSH into its oxidized form, glutathione disulfide, which is implicated in oxidative stress and its consequences [111,112].

Activation of ROS-associated enzymes and receptors by NPs is also involved in NP-induced generation of intracellular ROS. For example, metal oxide NPs (Ni_2_O_3_, Mn_2_O_3_, Co_3_O_4_, CoO, and Cr_2_O_3_ NPs) result in high level of oxidative-stress-mediated toxicity attributed to NADPH oxidation into NADP+, as well as cytochrome c oxidation [113]. This effect is correlated with band-gap energy levels associated with these NPs.

## 5. Biological Functions Modulated by NP-Induced ROS Production

The amount of ROS generated, and the resulting oxidative stress, are correlated with the nanomaterial concentration to which cells are exposed [84]. Cells exposed to low NP concentrations showed potent antioxidant defenses capable of overcoming oxidative stress and recovering the redox balance. By contrast, exposure to high NP concentrations overwhelms antioxidant systems and results in cytotoxicity and inflammation.

ROS elements, such as O_2_^•–^, HO^•^, and H_2_O_2_, are significant intermediates that are generated from physiological processes, including photosynthesis, respiration, and cell signaling, and their concentration inside cells is acutely regulated by enzymes, such as SOD, CAT, and GPX, or antioxidants, including ascorbic acid, cysteine, glutathione, and bilirubin [114]. Redox homeostasis can be disrupted as a result of numerous disorders, with oxidative stress representing ROS surges that can result in harm to cells via oxidative damage [115].

Oxidative stress is a key factor involved in nanotoxicity, as well as in alterations to cell motility, cytotoxicity, unregulated cell signaling, DNA damage, apoptosis, and cancer proliferations and metastasis [84,85,116]. The role of ROS in NP-induced biological functions in cells and the molecular mechanisms involved is outlined in the following subsections (Figure 2B).

### 5.1. DNA Damage and Cytotoxicity

The link between metallic NPs and chromosomal aberrations and oxidative damage to DNA was previously reported [117]. The potential of NPs to cause DNA damage can be attributed to the generation of the free radical HO^•^, which interacts with DNA to form 8-hydroxyl-2′-deoxyguanosine (8-OHdG) that ultimately leads to DNA damage [118]. In HO^•^^−^ mediated DNA damage, 8-OHdG is significantly increased during in vitro and in vivo exposure to NPs [119,120]. Interestingly, an in vivo study showed that exposure to Ag, Ti, Fe, or Cu NPs leads to nucleic acid damage-mediated genotoxicity [121].

At the beginning of ROS generation, oxidation of polyunsaturated fatty acids occurs, followed by production of lipid peroxides [122]. Lipid peroxidation-associated mutations are also implicated in metal NP-induced genotoxicity [123,124].

A combination of nanomaterials induce toxicity mediated by ROS in numerous biological systems, including skin fibroblasts, human erythrocytes, and different tumor cells [125]. The implication of oxidative-stress-mediated upregulation of key signaling pathways involved in activation of inflammatory factors, such as tumor necrosis factor-α and interleukins, was previously reported [34]. ROS is also involved in inflammatory responses that enhanced by metallic NPs (TiO_2_ NPs and SiO_2_ NPs) [126,127].

In human lung fibroblasts, AuNP exposure results in high levels of oxidative stress that occur simultaneous to the up-regulation of autophagy evident from increases in microtubule-associated protein 1 light-chain 3 (LC3) and autophagy gene 7 [128]. Adenosine monophosphate-treated human lung fibroblasts exhibited oxidative damage that provided evidence of malondialdehyde (MDA) protein adducts and increased expression of antioxidant genes. Autophagy is considered a protective mechanism against AuNP-induced cell toxicity.

ZnO NPs enhance cytotoxicity, which primarily occurs through ROS generation, which triggers oxidative injury and release of inflammatory mediators that ultimately lead to cell death in phagocytic RAW 264.7 cells and transformation in human bronchial epithelial BEAS-2B cells [85,129]. An Au-Co nanoalloy-induced alteration in tumor-initiating genes associated with an increase of micronuclei formation and generation of 8-OHdG was identified in mice as a result of increases in oxidative stress [130].

**Figure 3:**
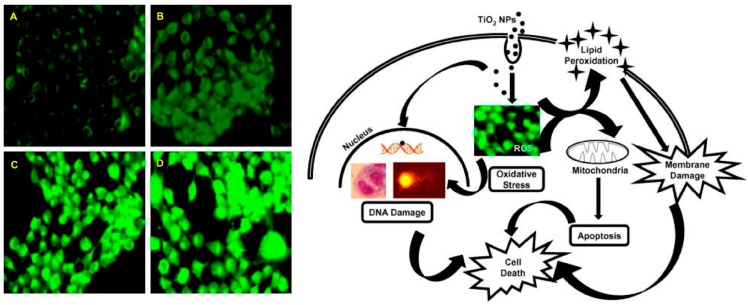
TiO_2_ NPs-induced ROS generation in human epidermal cells. Left panel showing 2′,7′-dichlorodihydrofluorescein diacetate (H_2_DCFDA) staining for dose-dependent ROS generation in TiO_2_ NPs-treated human epidermal cells (Magnification ×200). Right panel summarizing the role of ROS in TiO_2_-induced cell death in human epidermal cells. (**A**) Control-untreated cells; (**B**–**D**) dose-dependent exposure to TiO_2_ NPs. Right panel describing the proposed mechanism of ROS-mediated cytotoxicity in TiO_2_ NPs-treated human epidermal cells. (Reproduced from [123] with permission of Elsevier and Copyright Clearance Center).

In human epidermal keratinocytes, treatment of single-walled carbon nanotubes (SWCNTs) leads to cytotoxicity accompanied by oxidative stress indicated by detection of free radicals and increases in peroxidation [131]. In addition, TiO_2_ NP-exposed human epidermal cells showed oxidative stress-mediated genotoxicity, as presented by the formation of micronucleus and DNA degradation (Figure 3) [123]. These findings indicated that oxidative stress is a crucial mediator in the dermal toxicity of nanomaterials.

In NIH3T3 fibroblast cells, AgNP-induced apoptosis is correlated with ROS generation and activation of c-Jun N-terminal kinase (JNK) signaling [132]. AgNP-exposed NIH3T3 cells exhibited release of cytochrome c into the cytoplasm and detection of Bax in the mitochondria, indicating a role for mitochondria in AgNP-induced ROS generation. Suppression of both ROS and JNK signaling abrogated AgNP-induced apoptosis in NIH3T3 cells. Additionally, in mouse lymphoma cells, AgNPs stimulated mutations and oxidative stress facilitated by ROS formation [133]. Autophagy is a primary response to AgNP-induced oxidative stress in NIH3T3 cells, as indicated by up-regulation of LC3 and autophagosome formation [134].

### 5.2. Antimicrobial Function

**Figure 4:**
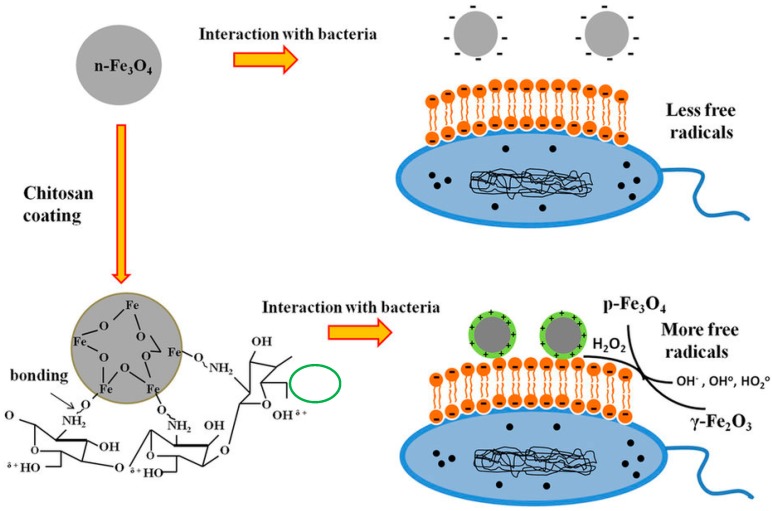
Diagram presenting the role of ROS production in the antimicrobial mechanism of IONPs against *Escherichia coli* and *Bacillus subtilis*. (Reproduced from [139], Copyright (2016) Creative Commons Attribution 4.0 International).

NP-induced oxidative stress can be exploited for killing a wide range of pathogens. Specially, the emergence of the antibiotic resistance in various bacteria, which hinder the efficiency of the antimicrobial therapy, has incited the need for discovery of new antibacterial mechanisms [135]. Several research reports evidenced the role of oxidative stress in NP-induced antimicrobial activity. Biologically-synthesized AgNPs showed potent antimicrobial activity against *Pseudomonas aeruginosa*, *Escherichia coli*, and *Staphylococcus aureus*. The significant antimicrobial effect of AgNP is attributed to its potential up-regulation of ROS and reactive nitrogen intermediates that eventually leads to killing of the bacteria [135]. ROS are involved in the significant antibacterial activity of ZnO NPs [136,137]. The role of singlet oxygen and hydroxyl radicals in the antifungal activity of ZnO NPs against *Candida albicans* has been proved [138]. Iron oxide NP(IONPs) that coated with the positively charged chitosan possesses special interface that showed significant production of ROS, which is involved in its significant antimicrobial activity against *Escherichia coli* and *Bacillus subtilis* (Figure 4) [139].

### 5.3. Cellular Differentiation

**Figure 5:**
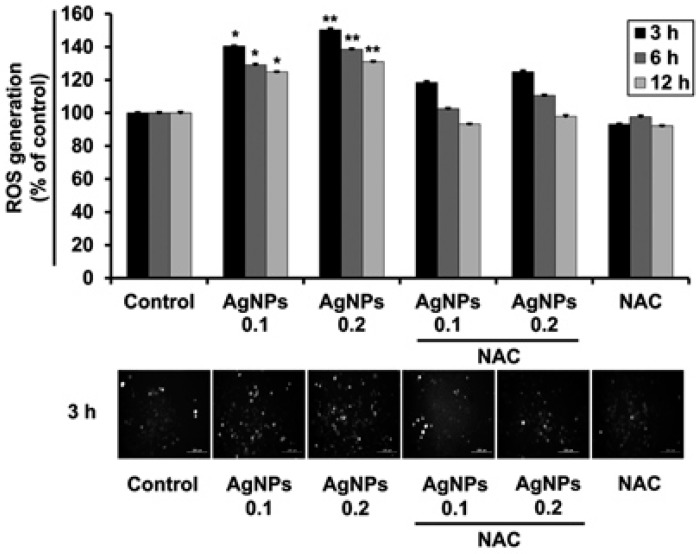
AgNP-exposed SH-SY5Y cells showed significant ROS production. Upper panel showing the readings of ROS level by spectrophotometer of dose-dependent treatment of AgNP. Lower panel presenting the fluorescent intensities of H_2_DCFDA staining (Scale bars, 200 μm). * *p* < 0.05; ** *p* < 0.01; and NAC: N-acetyl cysteine. (Reproduced from [140] with permission of Copyright Wiley-VCH Verlag GmbH and Co. KGaA).

NP-induced modulation of ROS generation plays important roles in cellular differentiation. Our research group showed the potential of biologically synthesized AgNPs to significantly generate ROS and to promote neurite growth in human neuroblastoma SH-SY5Y cells (Figure 5) [140].

In K562 cells, nitrogen-doped TiO_2_ NPs together with light energy of 12 J/cm^2^ (NP-based photodynamic therapy (PDT)) enhanced megakaryocytic terminal differentiation, which is mediated by autophagy [141]. Pharmacological inhibition of autophagy abrogated the effect of NP-based PDT to trigger the differentiation of K562 cells, and pretreatment with ROS-scavenging compounds suppressed the observed differentiation. These findings indicated that ROS is an upstream modulator of NP-based PDT-mediated terminal differentiation of megakaryocytes [141].

### 5.4. Anticancer

High levels of oxidative stress and aerobic glycolysis are hallmarks of tumor cells [142]. Oxidative stress is associated with abnormal growth of tumor cells, which is attributed to redox imbalance or disturbance of ROS-scavenging [143]. Additional oxidative stress from exposure to ROS-inducing agents leads to cell death due to toxicity induced by excess ROS production [144]. In this regard, the ability of NPs to generate ROS could potentially be exploited for cancer therapy.

In MCF-7 human breast cancer cells, Zn-doped TiO_2_ NP exposure leads to drastic decreases in cell viability and increased cell cycle arrest associated with increases in oxidative stress [145]. The oxidative stress induced in Zn-doped TiO_2_ NP-treated MCF-7 cells was indicated by depletion of GSH, decreases in SOD expression, and increases in the expression of the heme oxygenase-1 gene. This cytotoxicity was abrogated following treatment with *N*-acetyl-cysteine [145].

A previous study reported a role for p53 in cytotoxicity induced by ZnO NPs [146]. The exposure to low concentrations of ZnO NPs leads to upregulation of p53 that subsequently enhances the expression of antioxidant genes, including *ALDH4A1*, *GPX1*, *SOD2*, *SESN1*, and *SESN2*, whereas high concentrations of ZnO NPs result in the robust generation of ROS that trigger p53-induced apoptotic cell death. Cancer cells lacking p53, such as DLD-1 and SW480 cells, were more susceptible to toxicity induced by ZnO NPs [146].

The anticancer effect of NP-induced ROS generation can be attributed to apoptosis, necrosis, and autophagy. In MDA-MB-231 human breast cancer cells, AgNP exposure resulted in increased ROS generation and led to significant suppression of cell growth in a dose-dependent manner [147]. These cells also showed alterations in apoptosis evident in the activation of caspase-3.

SPION micelle-treated cells exhibited high levels of ROS generation concomitant with mitochondria-dependent apoptosis indicated by upregulation of caspase-3 and caspase-9, as well as high ratios of Bax/Bcl-2 [148]. The novel anticancer drug β-lapachone (β-lap) selectively suppresses the growth of cancer cells by up-regulating ROS generation. A recent study reported synergistic effects between SPION and β-lap in cancer treatment, where the authors showed that pretreatment of SPIONs prior to β-lap led to a ~10-fold increase in ROS generation relative to β-lap treatment alone, as well as increased therapeutic index of β-lap as a consequence of combination therapy [149]. This significant synergistic effect was attributed to the Fenton reaction that enhanced the release of Fe ions by SPION micelles inside the cancer cell to interact with H_2_O_2_ produced by β-lap. This activity was attenuated upon treatment with iron chelators. In human lung cancer cells, oxidative stress mediated cell death induced by exposure to copper oxide NPs [150,151].

## 6. NP-Induced Modulation of ROS Generation in Stem Cell Biology

The influence of metallic NPs on mesenchymal stem cell (MSC) toxicity has been investigated. A previous report illustrated toxic behavior of ZnO NPs in bone marrow MSCs (BM-MSCs) [152]. The acidic compartment housing lysosomal enzymes led to the instability of ZnO NPs and the subsequent release of Zn^+^. Additionally, BM-MSCs-exposed to ZnO NPs showed dose-dependent cytotoxicity, which correlated with ROS generation. Furthermore, activation of caspase-3/7 and alterations in apoptosis were observed in ZnO NP-treated BM-MSCs [152].

Recently, results indicated the potential of AgNPs to promote adipogenic differentiation of human MSCs (hMSCs) in an ROS-dependent mechanism [153]. AgNP-induced adipogenic differentiation was confirmed by the detection of lipid droplets and increases in the expression of adipogenic differentiation-related transcription factors. Moreover, the potent antibacterial activity of AgNPs enhanced hMSC adhesion. Interestingly, AgNPs did not show any negative influence on osteogenic differentiation of hMSCs [153]. Collectively, AgNPs could be a promising nanomaterial capable of exploiting ROS-related mechanisms involved in stem cell differentiation, as well as its potential for utilization in stem cell therapy and tissue engineering.

**Figure 6:**
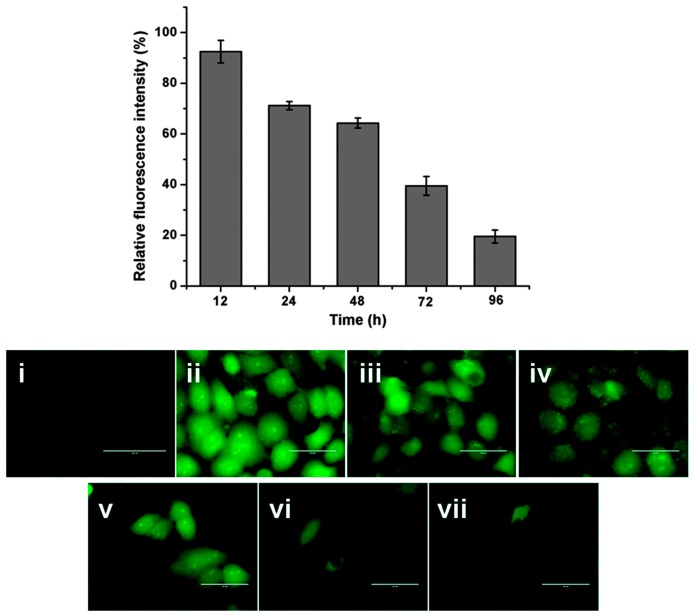
ROS scavenging potential of nanoceria in hydrogen peroxide H_2_O_2_-exposed L-132. (**i**) Control-untreated cell line; (**ii**) H_2_O_2_- exposed cells; (**iii**–**vii**) Time dependent BCNPs pretreatment in H_2_O_2_- exposed cells (Scale bars, 50 μm). (Reproduced from [158] with permission of The Royal Society of Chemistry).

Cerium (Ce) oxide NPs (nanoceria) are considered inorganic antioxidants capable of scavenging free radicals via similar mechanisms as those of SOD and CAT [154,155,156]. Ce^4+^ and Ce^3+^ species located on the surface of nanoceria are implicated in modulation of redox states [157]. The delivery of nanoceria via its encapsulation inside the biodegradable albumin NPs to finally form nanoceria encapsulated albumin nanoparticles (BCNPs) potently scavenged ROS production in hydrogen peroxide (H_2_O_2_)-exposed human lung epithelial cell line (L-132) and consequently prevented the apoptotic changes (Figure 6) [158]. Therefore, the antioxidant property of nanoceria could be exploited in the treatment and control of diseases caused by excessive ROS production.

In rat MSCs, nanoceria exposure leads to suppression of adipogenic differentiation via inhibition of ROS generation, which is essential for MSC differentiation into adipocytes [159]. These NPs could be promising nanomaterials for obesity therapy.

A recent study reported the toxic behavior of SPIONs in neural stem cells (NSCs) attributed to their potential to disturb redox balance and increase oxidative stress [160]. SPION-treated NSCs showed mitochondrial hyperpolarization, significant diminution in the level of intracellular GSH, cell membrane breakage, and changes in levels of GPX and SOD [160].

Another study investigated the effect of polysaccharide- and hydrocarbon-coated AgNPs [161]. Both of the chemically modified AgNPs exhibited negative effects on embryonic stem cell (ESC) proliferation and self-renewal, which were induced by AgNP-induced ROS production. ESC-treated AgNPs showed cell cycle arrest at the G1/S phase; however, polysaccharide-coated AgNPs showed less ROS generation, as well as less toxic effects, as compared with those shown by hydrocarbon-coated AgNPs [161].

## 7. Conclusions

Nanotechnology offers the potential for various biomedical applications regarding cancer therapy, drug delivery, imaging, and diagnosis and therapy of Alzheimer’s and cardiovascular diseases. Current advances in NP engineering indicate that well-designed nanomaterials have the potential to improve healthcare in the future. Manufacturing of engineered nanomaterials for commercial use has also grown exponentially. As a result, the safety and toxicity of nanomaterials have become a matter of increased public attention. In this review, we described the factors involved in NP-mediated ROS generation. We also highlighted the crucial roles of NP-induced ROS modulation on cellular functions, such as toxicity, anticancer activity, and cellular differentiation via modulation of a wide range of cellular signaling pathways. Additionally, NP-induced ROS production exhibits various functions in stem cell biology. The daily exposure of the humans to nanomaterials is inevitable. Moreover, the mechanisms associated with toxicity and the hazards involved in their use resulting in NP-induced ROS remain unclear. Therefore, further studies are needed to increase the understanding of mechanisms related to ROS generation by NP. This information will be aid in the modification of the physical and chemical properties of NPs to modulate the ROS generated, enabling NPs to be exploited in various medical applications and stem cell therapeutics.
